# Trends in microbiology testing in Australian long-term care facilities: national cohort study

**DOI:** 10.1186/s12879-026-13656-1

**Published:** 2026-06-01

**Authors:** Yohanes A. Wondimkun, Maria C. Inacio, Peter Lekkas, Catherine Lang, Noleen Bennett, Leon J. Worth, Karin Thursky, Malcolm Clark, Rodney James, Janet K. Sluggett

**Affiliations:** 1 School of Allied Health and Human Performance, College of Health, Adelaide University, Adelaide, South Australia Australia; 2 South Australian Health and Medical Research Institute, Adelaide, South Australia Australia; 3 Registry of Senior Australians Research Centre, South Australian Health and Medical Research Institute, Adelaide, South Australia Australia; 4 Registry of Senior Australians Research Centre, Caring Futures Institute, College of Nursing and Health Sciences, Flinders University, Bedford Park, South Australia Australia; 5 University of South Australia, UniSA Allied Health and Human Performance, Adelaide, South Australia Australia; 6 Victorian Healthcare Associated Infection Surveillance System (VICNISS) Coordinating Centre, Melbourne, VIC Australia; 7 Department of Infectious Diseases, National Centre for Antimicrobial Stewardship, The University of Melbourne, Parkville, VIC Australia; 8 Department of Nursing, Melbourne School of Health Sciences, The University of Melbourne, Parkville, VIC Australia; 9 Sir Peter MacCallum Department of Oncology, The University of Melbourne, Parkville, VIC Australia; 10 Department of Medicine, The University of Melbourne, Parkville, VIC Australia; 11 The Royal Melbourne Hospital Guidance Group, Melbourne Health, Melbourne, VIC Australia; 12 Department of General Practice, University of Melbourne, Parkville, VIC Australia

**Keywords:** Antimicrobial stewardship, Australia, Care homes, Diagnostic stewardship, Infectious disease, Long-term care facilities, Microbiology testing, Nursing homes, Older people

## Abstract

**Background:**

Microbiology testing can guide infectious disease management and antimicrobial selection, including for older people living in long-term care facilities (LTCFs), who are at high risk of infectious diseases. However, insufficient and excessive microbiology testing can contribute to inappropriate antimicrobial use. The study examined national trends, co-testing, and LTCF variation in microbiology testing.

**Methods:**

This national repeated cross-sectional study included individuals aged 65–105 years residing in Australian LTCFs between 2009 and 2019. The annual age and sex standardised (i) proportion of individuals with a microbiology test and (ii) number of tests performed/100 resident-years were determined. Annual changes were estimated using adjusted rate ratios (aRRs) with 95% confidence intervals (CIs) from Poisson or negative binomial models. Co-testing and LTCF variation in 2019 were also evaluated.

**Results:**

Among the 547,067 studied residents, the median age at study entry was 84 years and 65.8% were women. Proportion of microbiology testing increased from 50.2% (95%CI 49.8–50.5) in 2009 to 59.4% (95%CI 59.0-59.7) in 2019 (aRR 1.02, 95%CI 1.01–1.02). Urine tests were the most frequently requested microbiology test, increasing from 40.9% (95%CI 40.5–41.2) to 44.9% (95%CI 44.5–45.2) during 2009–2019 (aRR 1.01, 95%CI 1.01–1.01). Nucleic acid amplification tests (NAATs) increased from 3.4% (95%CI 3.3–3.5) to 17.9% (95%CI 17.7–18.1) (aRR 1.21, 95%CI 1.21–1.21). In 2019, there were 1,614 (58.6%) LTCFs where the adjusted proportion of microbiology testing fell within the 95%CI range around the population mean. Faecal, *Cryptosporidium/Giardia*, and *Clostridioides difficile/C.toxin* tests were commonly performed concurrently with NAATs.

**Conclusions:**

Over an 11-year period, microbiology testing increased. Eight out of ten residents were tested at least once during their LTCF stay, and considerable facility variation in testing was observed. Diagnostic stewardship targeting commonly requested microbiology tests (e.g., urine tests) could optimise antimicrobial use in LTCFs.

**Supplementary Information:**

The online version contains supplementary material available at 10.1186/s12879-026-13656-1.

## Introduction

Residents of long-term care facilities (LTCFs; also known as care homes or nursing homes) are at high risk of infectious diseases due to shared living arrangements, age-related physiological changes and underlying chronic health conditions [1, 2]. Infectious diseases remain a leading, yet potentially preventable cause of morbidity, hospitalisation and mortality in LTCFs [3–6]. A point prevalence survey of health care-associated infections (HALT-4) in 18 European countries, including 61,045 residents from 1,097 LTCFs, reported that 3% of residents had at least one suspected infection on the day of the survey [7]. Urinary tract, respiratory, gastrointestinal, and skin and soft tissue infections are the most common infections experienced in LTCFs [2, 7–9]. Antimicrobials are widely used in LTCFs, with up to 70% of residents dispensed at least one annually, with considerable variation observed across LTCFs [10, 11]. Furthermore, a high rate of broad-spectrum antimicrobial use, prolonged use for prophylaxis, and use without a documented indication occurs in LTCFs [7, 8]. 

Diagnosis and management of infectious diseases in LTCFs can be challenging due to factors such as difficulty in establishing an accurate clinical history, because of cognitive impairment, atypical clinical presentations (e.g., absence of fever), and limited access to infectious diseases physicians, and imaging and pathology services [12]. Diagnostic uncertainty, in a setting where local antibiograms are uncommon [13], can contribute to delayed or missed treatment, use of broad-spectrum antimicrobials that can increase the risk of adverse drug events, and antimicrobial resistance [14–16]. Microbiology testing can guide infectious disease management and treatment selection [17, 18]. However, point prevalence surveys have highlighted discordance between the rate of microbiology testing and antimicrobial use [7, 8]. For example, HALT-4 found that only 20% of suspected infections were confirmed through microbiology testing, while 68% had no examination [19]. Results were unknown in 9%, samples did not allow microorganism identification in 2%, and cultures were negative in 1% [19]. 

On the contrary, microbiology over-testing, including over-diagnosis of urinary tract infections (UTIs), may contribute to unnecessary antibiotic use [20]. Hence, there is a need to understand the extent of microbiology testing to inform targets for diagnostic stewardship programmes and support appropriate antimicrobial utilisation in LTCFs. Examining variation in microbiology testing across LTCFs, in a sector that has historically struggled with consistent delivery of high-quality care [11], helps identify potential gaps in practice and areas for improvement. However, very few population-level studies have examined the provision of microbiology testing in LTCFs [21], and none have examined co-testing or facility variation in microbiology testing at the national level in Australian LTCFs. This study aimed to examine annual trends, co-testing, and LTCF variation in microbiology testing.

## Materials and methods

### Study design, setting, data sources

We conducted a repeated cross-sectional study using data from the Registry of Senior Australians (ROSA) National Historical Cohort. ROSA is a national health and aged care data platform that links federal and state-based health, aged care and social welfare datasets to monitor the service utilisation and health outcomes of aged care recipients in Australia [22]. For this study, data sources accessed included the Australian Institute of Health and Welfare’s (AIHW) National Aged Care Data Clearinghouse (NACDC), which includes the National Death Index (NDI), and the Australian Government Medicare Benefits Schedule (MBS) and Pharmaceutical Benefit Scheme (PBS) datasets. The NACDC includes information on demographics, health conditions and care needs collected for individuals who had an aged care eligibility assessment and/or entry into LTCF assessment, and LTCF entry/exit dates. The NDI provides date and reason for death. The MBS dataset provides information on reimbursed claims for government-subsidised primary care services, including pathology tests, coded by MBS item codes [23]. The PBS dataset contains details of claims for government-subsidised prescription medicines dispensed via the PBS [24], coded by World Health Organization Anatomical Therapeutic Chemical (ATC) classification codes and PBS item codes [25]. 

### Study cohort

The study cohort comprised long-term residents aged 65–105 years, who did not identify as Aboriginal or Torres Strait Islander and resided in an Australian LTCF between 01/01/2009 and 31/12/2019 (Supplementary Fig. 1). Residents who self-identified as Aboriginal could not be included due to requirements of leadership from Aboriginal and specific Indigenous governance and ethics approvals, which were not part of our current study. Individuals who accessed healthcare services subsidised by the Department of Veterans’ Affairs were excluded due to known differences in primary care entitlements and service use [26]. Individuals who accessed permanent care for < 100 days were excluded in accordance with previous studies [10]. Included residents were followed until exiting a LTCF, 31 December 2019, or death, whichever came first. The final cohort included 547,067 individuals.

### Outcomes of interest

Primary outcomes of interest included annual age and sex standardised trends in (i) the proportion of individuals with at least one microbiology test and (ii) number of microbiology tests claimed per 100 resident-years. Co-ordering of microbiology tests and variation in testing across LTCFs were evaluated in the final study year (i.e., 2019). Microbiology tests were identified using relevant MBS item codes (excluding hospital inpatient claims) (Supplementary Table 1), and were evaluated overall and categorised as: body system (i.e., urine, skin/superficial, faecal, respiratory, ear, eye, nose and throat (EENT), genital, and blood); pathogen (i.e., *Cryptosporidium* spp. and *Giardia duodenalis*, *Clostridioides difficile* and *C. toxins*); and microbial nucleic acid amplification tests (NAATs) as per a previous study [27] and/or in line with current MBS code categories.

### Covariates

Covariates used to characterise the residents included: age at LTCF entry, sex, length of LTCF stay, country of birth (Australia, other), and comorbidity score at LTCF entry ascertained using Rx-Risk-V (a prescription-based comorbidity index applied to pharmaceutical claims in the six months before LTCF entry) [28]. Dementia and diabetes were ascertained from health condition information recorded in LTCF eligibility or entry assessments, or from the relevant Rx-risk categories [28]. History of long-term urinary catheter use (ongoing use), and care needs relating to skin/wound management were identified from LTCF entry assessments. Other covariates included the Australian state/territory of residence, LTCF ownership type (government, for-profit, not-for-profit) and remoteness (major city, outside major city) [29]. 

### Statistical analysis

Descriptive statistics (i.e., medians, interquartile ranges (IQRs), frequencies, proportions) were used to summarise characteristics of the overall cohort and annually. The proportion of residents with at least one microbiology test and number of tests claimed per 100 resident-days annually during 2009–2019 (overall and stratified by test type) were standardised for age and sex using direct standardisation. Annual changes in testing were examined using adjusted rate ratios (aRRs) with 95%CIs that were estimated using Poisson models or, if overdispersion was present, negative binomial models. Models were estimated with an autoregressive correlation structure and robust standard errors. Trends were also stratified by sex and the presence of dementia or diabetes, which could increase infection risk and impact microbiology testing [30]. 

To examine the co-testing pattern, the combination of microbiology tests (grouped by body system, pathogen, and technique of testing) claimed for the same individual on the same day, and within 7- and 14-day periods for the final year of our study (i.e., 2019) were visualised using heat maps. To examine national variation in testing during 2019, funnel plots were generated using adjusted estimates that were derived from logistic regression models, adjusting for age, sex, comorbidity score, and relevant health conditions (i.e., dementia, long-term urinary catheter, skin management, wound management). The 95%CIs around the population mean were estimated using the Wilson method [31]. Data from all individuals in the 2019 cohort contributed to the population mean estimate; however, facilities with < 20 residents were not shown to limit potential risk of reidentification. Data management and analyses were conducted using Stata (Release 18, TM: StataCorp LLC) and SAS software (version 9.4, SAS Institute Inc., Cary, NC, USA).

## Results

This study included 547,067 individuals, with the annual cohort size increasing from 147,972 in 2009 to 190,670 in 2019 (Table 1, Supplementary Table 2). The median age of the overall cohort was 84 years (IQR 79–89), and females accounted for two-thirds of those included (65.8%, *n* = 360,111). The median comorbidity score was 5 (IQR 3–7). Overall, over half of the residents (53.8%, *n* = 294,566) were living with dementia, while more than one in five (23.1%, *n* = 126,117) had diabetes.

**Table 1:** Characteristics of the study cohort, overall and in selected years

Characteristics	All residents 2009–2019	2009	2014	2019
Number of residents	547,067	147,972	168,484	190,670
Number of resident-days	529,317,727	42,221,283	47,534,233	54,906,744
Age at LTCF entry (years), median (IQR)	84 (79–89)	83 (78–88)	83 (78–88)	84 (78–88)
Female (n, %)	360,111 (65.8)	108,265 (73.2)	116,734 (69.3)	125,976 (66.1)
Born in Australia (n, %)	362,166 (66.3)	100,215 (67.8)	110,579 (65.7)	123,851 (65.2)
Missing (n)	1,078	183	118	746
Australian state/territory of residence (n, %)				
New South Wales	183,679 (33.6)	50,435 (34.1)	58,000 (34.4)	63,136 (33.1)
Victoria	146,143 (26.7)	39,200 (26.5)	44,463 (26.4)	51,306 (26.9)
Queensland	97,720 (17.9)	25,607 (17.3)	29,392 (17.4)	35,296 (18.5)
South Australia	51,214 (9.4)	14,569 (9.8)	15,981 (9.5)	17,104 (9.0)
Western Australia	46,369 (8.5)	12,537 (8.5)	14,312 (8.5)	16,436 (8.6)
Tasmania	14,578 (2.7)	3,862 (2.6)	4,179 (2.5)	4,668 (2.4)
Australian Capital Territory	6,382 (1.2)	1,494 (1.0)	1,836 (1.1)	2,381 (1.2)
Northern Territory	982 (0.2)	268 (0.2)	321 (0.2)	343 (0.2)
Remoteness of residence (n, %)				
Major city	380,738 (69.6)	103,758 (70.1)	118,584 (70.4)	134,306 (70.4)
Outside major city	166,329 (30.4)	44,214 (29.9)	49,900 (29.6)	56,364 (29.6)
Missing (n)	1,320	319	402	528
Comorbidity score and specific health conditions				
Rx risk comorbidity score, median (IQR)	5 (3–7)	5 (3–7)	5 (3–7)	5 (3–7)
Dementia (n, %)	294,566 (53.8)	86,811 (58.7)	87,405 (51.9)	96,501 (50.6)
Diabetes (n, %)	126,117 (23.1)	30,306 (20.5)	37,903 (22.5)	44,943 (23.6)
LTCF ownership type (n, %)				
Not-for-profit	302,800 (55.3)	86,807 (58.7)	95,822 (56.9)	104,568 (54.8)
For-profit	218,357 (39.9)	53,019 (35.8)	65,126 (38.7)	78,520 (41.2)
Government	25,910 (4.7)	8,146 (5.5)	7,536 (4.5)	7,582 (4.0)

Abbreviations: IQR - Interquartile range; LTCF - Long-term care facility

Between 2009 and 2019, the proportion of residents who were female decreased from 73.2% (*n* = 108,265) to 66.1% (*n* = 125,976), dementia prevalence decreased from 58.7% (*n* = 86,811) to 50.7% (*n* = 96,501), and those residing in for-profit LTCFs increased from 35.8% (*n* = 53,019 to 41.2% (*n* = 78,520) (Table 1, Supplementary Table 2). The prevalence of other characteristics studied remained steady.

### Proportion of individuals with at least one microbiology test annually

**Fig. 1:**
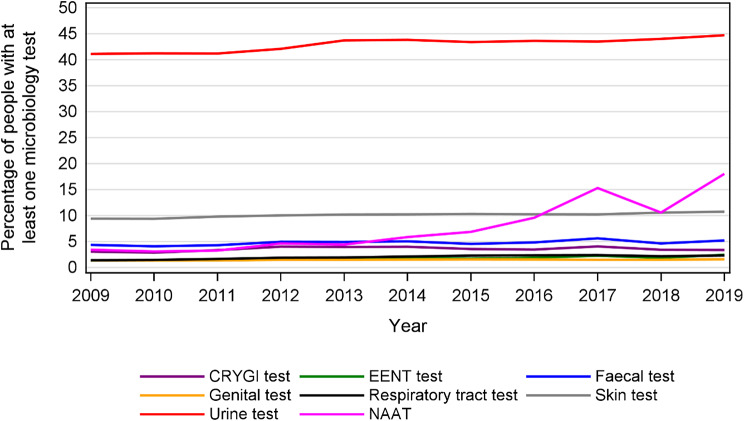
Crude trends in the proportion of individuals with at least one microbiology test annually among residents of Australian long-term care facilities between 2009 to 2019. Abbreviation: CRYGI - Cryptosporidium and Giardia; EENT- Eye, Ear, Nose, Throat; NAAT-nucleic acid amplification tests. Categories with < 1% were not presented in the figure for clarity

During the study period, 79.9% (95%CI 79.8–80.0) of residents had at least one microbiology test (Fig. 1; Table 2, Supplementary Table 3). Overall, the proportion of individuals with at least one microbiology test annually increased from 50.2% (95%CI 49.8–50.5) in 2009 to 59.4% (95%CI 59.0-59.7) in 2019, representing a 2% (aRR 1.02, 95%CI 1.01–1.02) increase annually. Urine tests were the most frequently performed microbiology test, increasing from 40.9% (95%CI 40.5–41.2) in 2009 to 44.9% (95%CI 44.5–45.2) in 2019, representing a 1% annual increment (aRR 1.01, 95%CI 1.01–1.01). Other commonly ordered tests included skin/superficial tests, which increased from 9.4% (95%CI 9.3–9.6) to 10.7% (95%CI 10.5–10.8) (aRR 1.01, 95%CI 1.01–1.01), while faecal testing increased from 4.3% (95%CI 4.2–4.4) to 5.2% (95%CI 5.1–5.3) (aRR 1.02, 95%CI 1.02–1.02). *Cryptosporidium/Giardia* tests were the most frequently requested pathogen-specific tests, with the incidence proportion increasing from 3.0% (95%CI 3.0-3.1) in 2009 to 3.4% (95%CI 3.3–3.5) in 2019 (aRR 1.01, 95%CI 1.01–1.01). Provision of NAATs increased from 3.4% (95%CI 3.3–3.5) in 2009 to 15.2% (95%CI 15.0-15.3) in 2017, then decreased to 10.5% (95%CI 10.3–10.6) in 2018, before increasing to 17.9% (95%CI 17.7–18.1) in 2019, representing overall a 21% annual increase (aRR, 1.21, 95%CI 1.21–1.21).

**Table 2:** Age and sex standardised proportion of individuals with at least one microbiology test annually (overall and specific tests) among older Australians in long-term care facilities, overall and in selected study years (%, 95% CI)

Microbiology tests	All residents (2009–2019)	2009	2014	2019	aRR, 95%CI
**Any microbiology tests**	79.9 (79.8–80.0)	50.2 (49.8–50.5)	54.1 (53.8–54.5)	59.4 (59.0-59.7)	1.02 (1.01–1.02)
**Tests grouped by body system**					
Urine	69.5 (69.4–69.7)	40.9 (40.5–41.2)	43.8 (43.5–44.1)	44.9 (44.5–45.2)	1.01 (1.01–1.01)
Skin/superficial	24.2 (24.1–24.3)	9.4 (9.3–9.6)	10.2 (10.1–10.4)	10.7 (10.5–10.8)	1.01 (1.01–1.01)
Faecal	13.9 (13.8–14.0)	4.3 (4.2–4.4)	5.0 (4.9–5.1)	5.2 (5.1–5.3)	1.02 (1.02–1.02)
Respiratory	5.4 (5.3–5.5)	1.4 (1.4–1.5)	2.1 (2.0-2.2)	2.3 (2.2–2.4)	1.04 (1.04–1.05)
Genital	4.3 (4.3–4.4)	1.3 (1.3–1.4)	1.5 (1.5–1.6)	1.6 (1.6–1.7)	1.02 (1.01–1.02)
EENT	5.3 (5.2–5.3)	1.4 (1.3–1.4)	1.8 (1.7–1.8)	2.4 (2.4–2.5)	1.06 (1.05–1.06)
Blood	2.0 (2.0-2.1)	0.5 (0.5–0.6)	0.6 (0.6–0.6)	0.7 (0.7–0.8)	1.03 (1.03–1.04)
**Tests for specific pathogens**					
*Cryptosporidium or Giardia*	10.6 (10.5–10.6)	3.0 (3.0-3.1)	4.0 (3.9–4.1)	3.4 (3.3–3.5)	1.01 (1.01–1.01)
*Clostridioides difficile or C.toxin*	0.4 (0.4–0.4)	0.1 (0.1–0.1)	0.2 (0.1–0.2)	0.2 (0.1–0.2)	1.06 (1.05–1.07)
**Other tests**					
NAAT	21.8 (21.7–21.9)	3.4 (3.3–3.5)	5.8 (5.7–5.9)	17.9 (17.7–18.1)	1.21 (1.21–1.21)

Abbreviations: aRR - Adjusted rate ratio; CI - Confidence interval; EENT - Eye, Ear, Nose, Throat; NAAT- Nucleic acid amplification tests

Stratification by sex found the crude proportion of individuals with at least one microbiology test annually was higher in females than males (61.3% vs. 56.7% in 2019) (Supplementary Fig. 2). Individuals without dementia had higher testing rates than people with dementia, while testing was also higher in people with diabetes. Similarly, a higher proportion of females received at least one urine test annually compared to males (47.4% vs. 40.7% in 2019) (Supplementary Fig. 3).

### Number of microbiology tests/100 resident-years

Overall, 196.3 (95% CI 195.6-196.9) tests/100 resident-years were conducted during the study period, with the number of tests increasing from 166.8 (95%CI 166.1-167.6) to 224.2 (95%CI 223.4–225.0) tests/100 resident-years between 2009 and 2019, representing a 3% annual increase (aRR 1.03, 95%CI 1.03–1.03) (Table 3, Supplementary Table 4). Trends in the volume of testing followed similar annual increases to the incidence proportions. Urine tests were the most performed tests, increasing from 118.4 (95%CI 117.8-119.1) to 141.1 (95%CI 140.5-141.7) between 2009 and 2019 (aRR 1.02, 95%CI 1.01–1.02). The number of *Cryptosporidium/Giardia* tests, the most common pathogen-specific test, increased from 4.5 (95%CI 4.4–4.6) to 4.9 (95%CI 4.8–5.1) tests/100 resident-years (aRR 1.01, 95%CI 1.01–1.01). NAATs increased from 5.9 (95%CI 5.7-6.0) to 30.4 (95%CI 30.1–30.7) tests/100 resident-years between 2009 and 2019 (aRR 1.21, 95%CI 1.21–1.21).

**Table 3:** Age and sex standardised number of microbiology tests/100 resident-years among older Australians in long-term care facilities, overall and specific tests, in selected study years (95% CI)

Microbiology tests	All residents (2009–2019)	2009	2014	2019	aRR, 95%CI
**Any microbiology test**	196.3 (195.6-196.9)	166.8 (166.1-167.6)	201.5 (200.7-202.3)	224.2 (223.4–225.0)	1.03 (1.03–1.03)
**Tests grouped by body system**					
Urine	133.0 (132.5-133.5)	118.4 (117.8-119.1)	138.9 (138.3-139.5)	141.1 (140.5-141.7)	1.02 (1.01–1.02)
Skin/superficial	19.9 (19.7–20.0)	18.4 (18.2–18.7)	20.6 (20.4–20.8)	20.6 (20.4–20.9)	1.01 (1.01–1.01)
Faecal	6.9 (6.9-7.0)	6.2 (6.1–6.3)	7.4 (7.2–7.5)	7.5 (7.4–7.6)	1.02 (1.02–1.02)
Respiratory	3.6 (3.5–3.6)	2.5 (2.4–2.6)	3.8 (3.7–3.9)	3.9 (3.8-4.0)	1.04 (1.04–1.05)
Genital	2.3 (2.2–2.3)	2.0 (1.9–2.1)	2.4 (2.3–2.4)	2.4 (2.4–2.5)	1.02 (1.02–1.02)
EENT	2.7 (2.7–2.7)	2.2 (2.1–2.3)	2.8 (2.7–2.9)	3.6 (3.5–3.7)	1.05 (1.05–1.05)
Blood	0.9 (0.9–0.9)	0.7 (0.7–0.8)	0.9 (0.8–0.9)	1.1 (1.0-1.1)	1.04 (1.03–1.04)
**Tests for specific pathogens**					
*Cryptosporidium or Giardia*	5.4 (5.3–5.4)	4.5 (4.4–4.6)	6.1 (5.9–6.2)	4.9 (4.8–5.1)	1.01 (1.01–1.01)
*Clostridioides difficile or C.toxin*	0.2 (0.2–0.2)	0.1 (0.1–0.1)	0.2 (0.2–0.3)	0.2 (0.2–0.2)	1.07 (1.05–1.08)
**Other tests**					
NAAT	13.4 (13.3–13.4)	5.9 (5.7-6.0)	9.8 (9.6–10.0)	30.4 (30.1–30.7)	1.21 (1.21–1.21)

Abbreviations: aRR - Adjusted rate ratio; CI - Confidence interval; EENT - Eye, Ear, Nose, Throat; NAAT- Nucleic acid amplification tests

### Microbiology tests co-performed on the same day, and the following seven and fourteen days

**Fig. 2:**
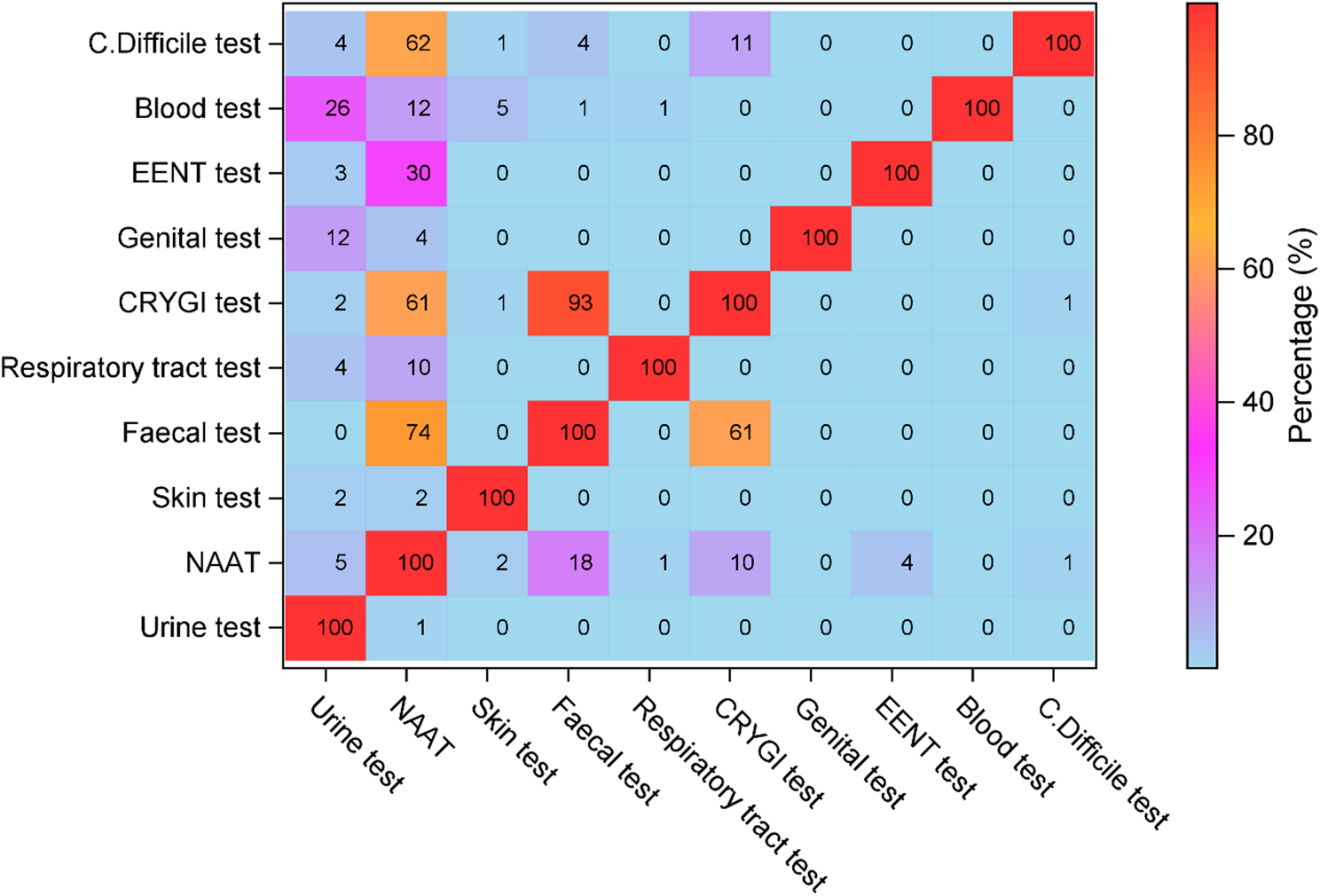
Heat map displaying the percentage of microbiology tests that were performed on the same day (shown in the column) as the microbiology test of interest (shown in the rows). Abbreviation: CRYGI - Cryptosporidium and Giardia; EENT - Eye, Ear, Nose, Throat; NAAT-Nucleic acid amplification tests. Note: this figure is read along the rows from left to right (e.g., 1% (2,104/211,565) of urine tests were conducted with a NAAT on the same day). Total number of each type of test in 2019: urine test (*n* = 211,565), NAATs (*n* = 55,630), skin/superficial tests (*n* = 31,340), respiratory tests (*n* = 5,979), faecal tests (*n* = 11,268), Cryptosporidium and Giardia (*n* = 7,433), genital tests (*n* = 3,578), EENT tests (*n* = 5,434), blood tests (*n* = 1,616), Clostridium difficile and C.toxin (*n* = 336)

Figure 2 shows the combinations of microbiology tests performed on the same day, with the prevalence of co-testing ranging from 0% to 93% depending on test type(s). Faecal, respiratory, EENT, blood, *Cryptosporidium/Giardia*, and *Clostridioides difficile/C.toxin*, tests were commonly performed concurrently with NAATs. *Cryptosporidium/Giardia* tests were commonly performed on the same day as faecal tests. The percentage in co-performed tests increased when the follow-up period was extended to seven and fourteen days, but there was no substantial change in the pattern (Supplementary Fig. 4).

### Facility variation in microbiology tests

**Fig. 3:**
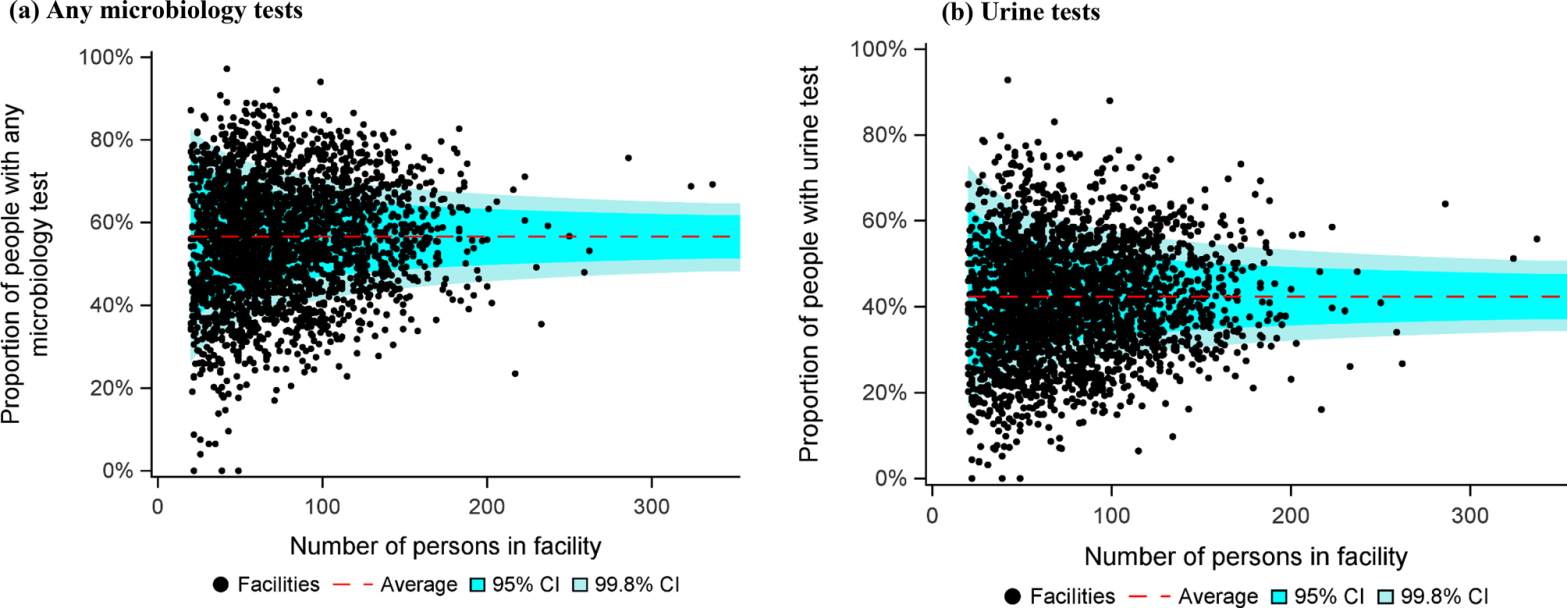
Funnel plots of long-term care facility variation in (**a**) any microbiology testing and (**b**) urine testing in 2019 (*n*=2,755 LTCFs)

The proportion of residents in a facility who received at least one microbiology test annually varied greatly across LTCFs (Fig. 3). There were 58.6% (*n* = 1,614) of LTCFs with an adjusted proportion of overall microbiology testing within the 95%CI range, with 20.0% (*n* = 551) sitting above and 21.4% (*n* = 590) below the upper and lower 95%CI limits, respectively (Supplementary Table 5). Similarly, 59.5% of LTCFs had an adjusted proportion of urine testing within the 95%CI range, with 17.4% (*n* = 478) sitting above and 23.2% (*n* = 639) below the 95%CI limits. LTCF variation in the adjusted proportion of residents who received faecal, skin/superficial, *Cryptosporidium/Giardia*, and NAATs was also observed (Supplementary Fig. 5).

## Discussion

This national population-based study of 547,067 residents from 3,484 LTCFs, found 80% of residents had at least one microbiology test during their LTCF stay. The proportion of residents who received at least one test increased 2% annually over the 11-year study period, from 50% in 2009 to 59% in 2019. Urine tests were the most common test, with 70% of residents tested during their LTCF stay. Our finding of 196 microbiology tests/100 resident-years suggests an equivalent of nearly two tests performed per resident per year during the study period. NAATs were often performed concurrently with other microbiology tests. The high rates of *Cryptosporidium/Giardia* testing on the same day as other faecal tests was an expected finding, given these tests are performed on stool samples. There was also considerable LTCF variation in testing, with 41.4% of LTCFs sitting outside the 95%CI range around the population mean. The increased testing is therefore underpinned by a high rate of urine testing, which is concerning and may reflect potential over-testing for UTIs in LTCFs. The considerable variation in testing across LTCFs suggests potential differences in the implementation of infection prevention and control (IPC) measures and antimicrobial stewardship, and access to healthcare providers and pathology services, suggesting potential for improvement.

The increased microbiology testing observed in the current study is in disagreement with a US Veterans Affairs LTCF study that reported 8% annual decreases in culture tests per admission (from 1.6 to 0.9 during 2010–2017) [21]. Because that study examined microbiology cultures only, it is unclear whether other types of tests (e.g., microscopy/molecular tests) followed the same trend. A point-prevalence survey of infectious diseases [7, 8] and a study examining infection-related hospitalisations from LTCFs reported very low rates of microbiology testing [16]. However, due to their cross-sectional assessment, these studies do not provide a comprehensive picture of microbiology testing practices over time. Educating care providers (e.g., when to request a urine culture), and using standardised resident assessments and decision-support tools may help to avoid unnecessary testing and overtreatment of presumed infections, while also minimising empiric antibiotic use due to undertesting [32–35]. 

Urine testing was the most common type of test performed. This is consistent with an Australian study of 9 LTCFs that examined positive culture results, finding that 70% of positive cultures originated from urine samples [13]. Similarly, in a UK primary care study, urine tests accounted for ≥ 73% of microbiology tests among people aged ≥ 65 years, or more depending on their age group [27]. Urine testing in the absence of UTI signs and symptoms, or when non-specific signs and symptoms are present (e.g., changes in urine appearance and volume, abdominal pain, falls, abnormal vital signs) may prompt unnecessary antibiotic use due to the presence of asymptomatic bacteriuria [20, 36, 37]. Urine culture, urinalysis and dipstick tests can not differentiate between a UTI and asymptomatic bacteriuria [37, 38]. As such, dipstick testing is not required to confirm a UTI diagnosis, while urine culture remains the standard test to confirm a diagnosis in the presence of signs and symptoms and determine antibiotic sensitivity [38–41]. Following the recommended clinical pathway during assessment of suspected UTI can aid judicious testing and minimise unnecessary antibiotic treatment [38, 41, 42]. 

NAATs were commonly performed and testing substantially increased during our study period, likely reflecting infrastructure advances in laboratories [43]. NAAT provision temporarily declined in 2018 before increasing again in 2019, which may be related to the MBS Review Taskforce’s suggestion around this period to restructure generic molecular items (e.g., MBS item number 69496) into system-specific MBS items [44]. Further, in our study, concurrent testing (i.e., tests conducted on the same day, following 7 or 14 days) varied depending on the test type, with faecal, *Cryptosporidium/Giardia* and *C. difficile* tests largely performed with NAATs [27]. Concurrent use of NAATs with traditional methods (e.g., culture-based) can enable the provision of effective and targeted antimicrobial treatment, thereby decreasing empirical selection, particularly in LTCFs where timely ordering of pathology tests is challenging and delays in receiving results are common [45, 46]. However, their high sensitivity means false positive results are possible with microbial contamination and colonisation, due to residual nucleic acid being present after a resolved infection, which could possibly lead to unnecessary antimicrobial use [46, 47]. This warrants a need to select the most appropriate test cautiously, and to consider clinical factors and additional investigations when using NAAT to diagnose infections and initiating antimicrobials [17, 45]. 

The considerable facility variation in testing observed in our study is potentially unwarranted, and is consistent with previous studies demonstrating variation in antibiotic use across Australian LTCFs [48–50]. In Australian, pathology services, including microbiology testing, are provided through approximately 500 public and private organisations, although the service delivery is highly concentrated among a few large providers [51, 52]. Public pathology providers primarily serve public hospitals, while private providers serve the community. LTCFs typically use external pathology providers, with the specimens collected on-site by qualified staff and transported to an off-site laboratory for analysis [53]. Variation in accessibility of pathology services, transportation, and turnaround time for results can also be a source of variation observed across LTCFs, particularly across non-metropolitan areas [16]. The most recent European HALT-4 point-prevalence survey also reported high variation in testing across countries, with prevalence of microbiology testing among residents with suspected infection ranging from 24% in Germany to 90% in the Netherlands [7]. Additional potential contributing factors include differences in the implementation of IPC measures and antimicrobial stewardship, variation in access to primary care/specialist providers, including infectious disease physicians (who do not practice in on-site Australian LTCFs) [49, 54]. Differences in access to hospitalisation avoidance programs, which could decrease infection-related hospitalisations but increase microbiology testing within LTCFs, may have also contributed [55]. Qualitative investigation into factors driving variation in microbiology testing is recommended.

Overall, the increases in microbiology testing (including high volumes of urine testing), concurrent use of NAATs, and facility variation suggest potential for improvement [33]. However, it must be considered that testing is also dependent on the specific equipment or other processing or billing decisions made in laboratories, which may lead to different tests being claimed compared to those on the clinician’s initial request form. In light of previously reported high and variable antibiotic use in LTCFs, these findings underscore the need to further investigate microbiology testing alongside antimicrobial use in LTCFs [10, 49]. A study examining microbiology testing around the time of antibiotic initiation (i.e., within − 14 to + 7 days) in the general Australian population found that only 19% of the antibiotic prescriptions were accompanied by microbiology testing [56]. Implementation of diagnostic stewardship in LTCFs (i.e., improved ordering, performing and/or reporting of tests) can complement antimicrobial stewardship efforts and improve healthcare resource use [34, 57]. While these multidisciplinary programs can be resource-intensive [47], targeted stewardship programs (e.g., focusing on urine or molecular tests) may be feasible [17, 34]. For example, ‘to dip or not to dip’ programs in LTCFs are feasible and effective [58, 59]. Similarly, a LTCF quality improvement program focused on increasing awareness of UTI guidelines showed decreases in urine cultures and diagnosis [60]. Broader implementation of effective programs is likely to improve infectious disease management and antimicrobial use in LTCFs. However, the success of these interventions is context-dependent and likely requires a coordinated approach that includes strengthening staff training and dedicated IPC, antimicrobial and diagnostic stewardship teams that are supported by evidence-based, LTCF-specific guidance [8, 61, 62]. The diagnostic process and antibiotic use could also be improved through use of validated quality indicators focusing on the most prevalent infections in LTCFs (e.g., monitoring of urinalysis, and antibiotic use without UTI-specific symptoms) [63, 64]. Since our study period, all Australian LTCFs are required to have a dedicated IPC lead and can access funding for an on-site clinical pharmacist, the impacts of which require further evaluation.

### Strengths and limitations

This national population-based cohort study of microbiology testing in LTCFs covering 11 years is representative of the LTCF population. We implemented various analysis approaches to ensure robustness of the findings, including examining the testing practice annually to limit the impacts of seasonal variation in testing (e.g., for respiratory infections) and exclusion of in-hospital tests to reflect the microbiology testing practice of LTCFs. We also examined co-testing, providing insight into the diagnostic practice and types of tests commonly co-initiated. Limitations include the inability to examine test results or resulting clinical decisions as these were not available within ROSA. Future studies could examine the timing of microbiology testing in relation to antibiotic initiation. Another limitation is that the MBS, which subsidises pathology testing, only allows the three most expensive pathology tests to be claimed on the same day [65]. Hence, under-ascertainment of certain tests is possible. We were also unable to assign NAATs to a specific body system or pathogen, resulting in heterogeneous tests being grouped in a single category, hence limiting its interpretability [66]. Moreover, any on-site testing, such as urine dipsticks, and imaging studies were not assessed. Finally, we analysed the most up-to-date data in ROSA at the time of data extraction and were therefore unable to examine the COVID-19 pandemic period, where testing trends may have changed and new tests were introduced [67]. 

## Conclusion

Eight out of ten residents had at least one microbiology test during their LTCF stay, with increasing annual trends during 2009–2019. Urine tests were the most commonly requested test, indicating a potential opportunity to review testing practices and associated antibiotic use. The frequent co-ordering of NAATs with other tests warrants further investigation. There were notable variations in microbiology testing across LTCFs, suggesting potential for improvements in infection prevention, diagnosis and management and the potential value of implementing targeted diagnostic stewardship programs in LTCFs.

## Supplementary Information

Below is the link to the electronic supplementary material.

Supplementary Material 1

## Data Availability

These data were made available to the researchers under ethical, governance, and confidentiality agreements that do not allow public sharing.

## Abbreviations

ATC: Anatomical Therapeutic Chemical
AIHW: Australian Institute of Health and Welfare’s
LTCF: Long-term care facility
NAAT: Nucleic acid amplification tests
NACDC: National Aged Care Data Clearinghouse
NDI: National Death Index
MBS: Medicare Benefits Schedule
PBS: Pharmaceutical Benefit Scheme
ROSA: Registry of Senior Australians
SAHMRI: South Australian Health and Medical Research Institute
UTIs: Urinary tract infections
