# Supplementary material for: Trends in microbiology testing in Australian long-term care facilities: national cohort study
Source: BMC Infect Dis. 2026 Jun 1;26:1394. doi: 10.1186/s12879-026-13656-1 (PMC13412369; doi:10.1186/s12879-026-13656-1)
Supplement: Supplementary file 1 — Supplementary Material 1 [file 12879_2026_13656_MOESM1_ESM.docx]

**Supplementary file**

**Supplementary Tables 1. List of Medicare benefit schedule (MBS) item number for microbiology tests**

| **Category** | **MBS Item numbers** | **MBS Item Descriptions** |
| --- | --- | --- |
| **Urine** | 69333 | Urine examination (including serial examinations) by any means other than simple culture by dip slide. |
|  | 73805, 73832 | Microscopy of urine, excluding dipstick testing.  Microscopy of urine, excluding dipstick testing by a participating nurse practitioner. |
| **Skin/superficial** | 69306 | Microscopy and culture to detect pathogenic micro-organisms from skin or other superficial sites; 1 or more tests on 1 or more specimens. |
|  | 69309 | Microscopy and culture to detect dermatophytes and other fungi causing cutaneous disease from skin scrapings, skin biopsies, hair and nails (excluding swab specimens); 1 or more tests on 1 or more specimens. |
|  | 73810, 73837 | Microscopy for fungi in skin, hair or nails -1 or more sites / Microscopy for fungi in skin, hair or nails by a participating nurse practitioner; 1 or more sites |
| **Faecal** | 69345 | Culture and (if performed) microscopy without concentration techniques of faeces for faecal pathogens, using at least 2 selective or enrichment media and culture in at least 2 different atmospheres; 1 examination in any 7-day period. |
| **Respiratory** | 69318 | Microscopy and culture to detect pathogenic micro-organisms from specimens of sputum; 1 or more tests on 1 or more specimens. |
| **Genital** | 69312 | Microscopy and culture to detect pathogenic micro-organisms from urethra, vagina, cervix, or rectum (except for faecal pathogens); 1 or more tests on 1 or more specimens. |
| **Eye/Ear/Nose/Throat (EENT)** | 69303 | Culture and (if performed) microscopy to detect pathogenic micro-organisms from nasal swabs, throat swabs, eye swabs and ear swabs (excluding swabs taken for epidemiological surveillance), including (if performed):  (a) pathogen identification and antibiotic susceptibility testing; or  (b) a service described in item 69300; specimens from 1 or more sites |
| **Blood** | 69354, 69357, 69360 | Blood culture for pathogenic micro-organisms (other than viruses), including sub-cultures and (if performed): (a) identification of any cultured pathogen; and (b) necessary antibiotic susceptibility testing; to a maximum of 3 sets of cultures |
| **Targeted** |  |  |
| *Cryptosporidium/Giardia* | 69336**,** 69339 | Microscopy of faeces for ova, cysts and parasites that must include a concentration technique, and the use of fixed stains or antigen detection for cryptosporidia and giardia. |
| *C diff/ C diff toxin* | 69363 | Detection of Clostridium difficile or Clostridium difficile toxin (except if a service described in item 69345 has been performed); one or more tests. |
| *Other tests* |  |  |
| **Microbial nucleic acid amplification tests** | 69364, 69365, 69367, 69372, 69373, 69374, 69375, 69376  69494, 69495, 69496, 69497, 69498 | Detection of a virus or microbial antigen or microbial nucleic acid (not elsewhere specified);1 or more tests (Item is subject to rule 6 and 26) |

**The following tests were also included in the overall testing category but were not reported individually due to very low prevalence in the LTCF cohort:** *Mycobacteria (*MBS item numbers 69324, 69325, 69327, 69328, 69330, 6933), *Chlamydia trachomatis (*69315, 69369, 69370, 69316, 69317, 69319), *hepatitis virus (*69444,69445, 69451, 69475, 69478, 69481, 69482, 69483, 69484, 69488, 69489, 69491, 69492, 69499, 69500, 69472, 69474), *Epstein Barr Virus (*69472, 69474), 69300,69321, 73807, 73834, 73808, 73835 (microscopy testing for tests not classified elsewhere).

**Supplementary Table 2. Characteristics of the study cohort, overall and yearly**

| Characteristics | 2009-19 | 2009 | 2010 | 2011 | 2012 | 2013 | 2014 | 2015 | 2016 | 2017 | 2018 | 2019 |
| --- | --- | --- | --- | --- | --- | --- | --- | --- | --- | --- | --- | --- |
| Number of residents | 547,067 | 147,972 | 152,677 | 156,718 | 160,567 | 163,569 | 168,484 | 173,737 | 178, 976 | 184, 608 | 188,584 | 190,670 |
| Number of resident-days | 529,317,727 | 42,221,283 | 43,405,567 | 44,431,509 | 45,396,053 | 46,379,658 | 47,534,233 | 48,743,251 | 50,387,654 | 51,782,447 | 53,542,287 | 54,906,744 |
| Age at LTCF entry,  median (IQR) | 84 (79-89) | 83 (78-88) | 83 (78-88) | 83 (78-88) | 83 (78-88) | 83 (78-88) | 83 (78-88) | 83 (78-88) | 84 (78-88) | 84 (78-88) | 84 (78-88) | 84 (78-88) |
| Female (n, %) | 360,111 (65.8) | 108,265 (73.2) | 110,871 (72.6) | 112,476 (71.8) | 114,040 (71.0) | 114,697 (70.1) | 116,734 (69.3) | 118,951 (68.5) | 121,025 (67.6) | 123,671 (67.0) | 125,421 (66.5) | 125,976 (66.1) |
| Born in Australia | 362,166 (66.3) | 100,215 (67.8) | 102,811 (67.4) | 104,668  (66.9) | 106,460  (66.4) | 107,951 (66.0) | 110,579 (65.7) | 113,660 (65.5) | 116,878 (65.3) | 120,608 (65.4) | 122,750 (65.2) | 123,851 (65.2) |
| Missing (n) | 1,078 | 183 | 156 | 153 | 140 | 127 | 118 | 104 | 108 | 223 | 455 | 746 |
| Australian state/territory of residence (n, %) |  |  |  |  |  |  |  |  |  |  |  |  |
| New South Wales | 183,679 (33.6) | 50,435 (34.1) | 52,016  (34.1) | 53,702 (34.3) | 55,150 (34.3) | 56,303 (34.4) | 58,000 (34.4) | 59,236 (34.1) | 60,829 (34.0) | 62,415 (33.8) | 63,202 (33.5) | 63,136 (33.1) |
| Victoria | 146,143 (26.7) | 39,200 (26.5) | 40,413  (26.5) | 41,188 (26.3) | 41,971 (26.1) | 42,883 (26.2) | 44,463 (26.4) | 46,371 (26.7) | 47,821 (26.7) | 49,512 (26.8) | 50,657 (26.9) | 51,306 (26.9) |
| Queensland | 97,720 (17.9) | 25,607 (17.3) | 26,727  (17.5) | 27,451 (17.5) | 28,159 (17.5) | 28,602 (17.5) | 29,392 (17.4) | 30,492 (17.6) | 31,702 (17.7) | 33,216 (18.0) | 34,324 (18.2) | 35,296 (18.5) |
| South Australia | 51,214 (9.4) | 14,569 (9.8) | 14,785  (9.7) | 15,057 (9.6) | 15,469 (9.6) | 15,640 (9.6) | 15,981 (9.5) | 16,360 (9.4) | 16,690 (9.3) | 17,011 (9.2) | 17,177 (9.1) | 17,104 (9.0) |
| Western Australia | 46,369 (8.5) | 12,537 (8.5) | 12,962  (8.5) | 13,334 (8.5) | 13,652 (8.5) | 13,977 (8.5) | 14,312 (8.5) | 14,620 (8.4) | 15,063 (8.4) | 15,270 (8.3) | 15,884 (8.4) | 16,436 (8.6) |
| Tasmania | 14,578 (2.7) | 3,862 (2.6) | 3,938  (2.6) | 4,003 (2.6) | 4,114 (2.6) | 4,099 (2.5) | 4,179 (2.5) | 4,350 (2.5) | 4,366 (2.4) | 4,583 (2.5) | 4,627 (2.5) | 4,668 (2.4) |
| Australian Capital territory | 6,382 (1.2) | 1,494 (1.0) | 1,568(1.0) | 1,676 (1.1) | 1,724 (1.1) | 1,734 (1.1) | 1,836 (1.1) | 1,987 (1.1) | 2,195 (1.2) | 2,298 (1.2) | 2,370 (1.3) | 2,381 (1.2) |
| Northern Territory | 982 (0.2) | 268  (0.2) | 268  (0.2) | 307 (0.2) | 328  (0.2) | 331  (0.2) | 321 (0.2) | 321 (0.2) | 310  (0.2) | 303  (0.2) | 343  (0.2) | 343  (0.2) |
| Remoteness of residence (n, %) |  |  |  |  |  |  |  |  |  |  |  |  |
| Major city | 380,738 (69.6) | 103,758 (70.1) | 107,187 (70.2) | 109,909 (70.1) | 112,540 (70.1) | 114,878 (70.2) | 118,584 (70.4) | 121,804 (70.1) | 125,435 (70.1) | 129,439 (70.1) | 132,410 (70.2) | 134,306 (70.4) |
| Outside major city | 166,329 (30.4) | 44,214 (29.9) | 45,490 (29.8) | 46,809 (29.9) | 48,027 (29.9) | 48,691 (29.8) | 49,900 (29.6) | 51,933 (29.9) | 53,541 (29.9) | 55,169 (29.9) | 56,174 (29.8) | 56,364 (29.6) |
| Missing (n) | 1,320 | 319 | 329 | 330 | 348 | 394 | 402 | 404 | 433 | 476 | 527 | 528 |
| Rx risk comorbidity score, median (IQR) | 5(3-7) | 5(3-7) | 5(3-7) | 5(3-7) | 5(3-7) | 5(3-7) | 5(3-7) | 5(3-7) | 5(3-7) | 5(3-7) | 5(3-7) | 5(3-7) |
| Health conditions |  |  |  |  |  |  |  |  |  |  |  |  |
| Dementia (n, %) | 294,566 (53.8) | 86,811 (58.7) | 86,490 (56.6) | 86,368  (55.1) | 86,302 (53.7) | 86,301 (52.8) | 87,405 (51.9) | 89,232 (51.4) | 91,418 (51.1) | 94,027 (50.9) | 95,793 (50.8) | 96,501 (50.6) |
| Diabetes (n,%) | 126,117 (23.1) | 30,306 (20.5) | 31,863 (20.9) | 33,373  (21.3) | 34,822 (21.7) | 36,102 (22.1) | 37,903 (22.5) | 39,800 (22.9) | 41,579 (23.2) | 43,123 (23.4) | 44,056 (23.4) | 44,943 (23.6) |
| Facility type of residence (n, %) |  |  |  |  |  |  |  |  |  |  |  |  |
| Not for profit | 302,800 (55.3) | 86,807 (58.7) | 89,181 (58.4) | 90,891 (58.0) | 92,497 (57.6) | 94,192 (57.6) | 95,822 (56.9) | 97,619 (56.2) | 100,115 (55.9) | 102,688 (55.6) | 104,009 (55.2) | 104,568 (54.8) |
| For profit | 218,357 (39.9) | 53,019 (35.8) | 55,501 (36.4) | 57,862 (36.9) | 60,171 (37.5) | 61,734 (37.7) | 65,126 (38.7) | 68,461 (39.4) | 71,236 (39.8) | 74,282 (40.2) | 76,868 (40.8) | 78,520 (41.2) |
| Government | 25,910 (4.7) | 8,146 (5.5) | 7,995 (5.2) | 7,965 (5.1) | 7,899 (4.9) | 7,643 (4.7) | 7,536 (4.5) | 7,657 (4.4) | 7,625 (4.3) | 7,638 (4.1) | 7,707 (4.1) | 7,582 (4.0) |

**LTCF**-Long-term care facilities; **IQR**-Interquartile range

**Supplementary Table 3. Age and sex standardised proportion of overall microbiology testing, and provision of specific tests among older Australians in long-term care facilities, overall and annually between 2009 and 2019 (%, (95%CI))**

| **Microbiology tests** | **2009-19** | **2009** | **2010** | **2011** | **2012** | **2013** | **2014** | **2015** | **2016** | **2017** | **2018** | **2019** | **aRR 95%CI** |
| --- | --- | --- | --- | --- | --- | --- | --- | --- | --- | --- | --- | --- | --- |
| Any microbiology tests | 79.9 (79.8-80.0) | 50.2 (49.8-50.5) | 50.1 (49.8-50.5) | 50.6 (50.2-50.9) | 52.1 (51.7-52.5) | 53.5 (53.2-53.9) | 54.1 (53.8-54.5) | 54.1 (53.8-54.5) | 55.2 (54.9-55.5) | 57.5 (57.1-57.8) | 56.0 (55.6-56.3) | 59.4 (59.0-59.7) | 1.02 (1.01-1.02) |
| **Tests grouped by body system** | | | | | | | | | | | | | |
| Urine | 69.5 (69.4-69.7) | 40.9 (40.5-41.2) | 41.1 (40.7-41.4) | 41.1 (40.7-41.4) | 42.0 (41.7-42.3) | 43.7 (43.4-44.0) | 43.8 (43.5-44.1) | 43.4 (43.1-43.7) | 43.7 (43.4-44.0) | 43.6 (43.3-43.9) | 44.1 (43.8-44.4) | 44.9 (44.5-45.2) | 1.01 (1.01-1.01) |
| Skin/superficial | 24.2 (24.1-24.3) | 9.4 (9.3-9.6) | 9.4 (9.2-9.6) | 9.8 (9.7-10.0) | 10.0 (9.9-10.2) | 10.2 (10.0-10.4) | 10.2 (10.1-10.4) | 10.3 (10.1-10.4) | 10.2 (10.1-10.4) | 10.2 (10.0-10.3) | 10.5 (10.4-10.7) | 10.7 (10.5-10.8) | 1.01 (1.01-1.01) |
| Faecal | 13.9 (13.8-14.0) | 4.3 (4.2-4.4) | 4.1 (4.0-4.2) | 4.3 (4.2-4.4) | 4.9 (4.8-5.0) | 4.9 (4.8-5.0) | 5.0 (4.9-5.1) | 4.5 (4.4-4.6) | 4.8 (4.7-4.9) | 5.6 (5.5-5.7) | 4.7 (4.6-4.8) | 5.2 (5.1-5.3) | 1.02 (1.02-1.02) |
| Respiratory | 5.4 (5.3-5.5) | 1.4 (1.4-1.5) | 1.5 (1.4-1.5) | 1.7 (1.6-1.7) | 1.9 (1.8-2.0) | 1.9 (1.9-2.0) | 2.1 (2.0-2.2) | 2.3 (2.2-2.4) | 2.3 (2.3-2.4) | 2.4 (2.3-2.4) | 2.2 (2.1-2.2) | 2.3 (2.2-2.4) | 1.04 (1.04-1.05) |
| Genital | 4.3 (4.3-4.4) | 1.3 (1.3-1.4) | 1.3 (1.3-1.4) | 1.4 (1.3-1.4) | 1.5 (1.4-1.5) | 1.5 (1.4-1.5) | 1.5 (1.5-1.6) | 1.6 (1.5-1.6) | 1.6 (1.5-1.6) | 1.5 (1.5-1.6) | 1.5 (1.5-1.6) | 1.6 (1.6-1.7) | 1.02 (1.01-1.02) |
| EENT | 5.3 (5.2-5.3) | 1.4 (1.3-1.4) | 1.4 (1.3-1.4) | 1.4 (1.3-1.4) | 1.5 (1.5-1.6) | 1.6 (1.5-1.6) | 1.8 (1.7-1.8) | 1.7 (1.7-1.8) | 1.9 (1.8-1.9) | 2.3 (2.2-2.3) | 1.8 (1.8-1.9) | 2.4 (2.4-2.5) | 1.06 (1.05-1.06) |
| Blood | 2.0 (2.0-2.1) | 0.5 (0.5-0.6) | 0.5 (0.5-0.6) | 0.6 (0.6-0.6) | 0.6 (0.6-0.6) | 0.6 (0.6-0.6) | 0.6 (0.6-0.6) | 0.7 (0.6-0.7) | 0.6 (0.6-0.7) | 0.7 (0.7-0.8) | 0.7 (0.7-0.8) | 0.7 (0.7-0.8) | 1.03 (1.03-1.04) |
| **Tests targeting specific pathogens** | | | | | | | | | | | | | |
| *Cryptosporidium & Giardia* | 10.6 (10.5-10.6) | 3.0 (3.0-3.1) | 2.9 (2.8-2.9) | 3.3 (3.2-3.4) | 4.0 (3.9-4.1) | 3.9 (3.8-4.0) | 4.0 (3.9-4.1) | 3.6 (3.5-3.7) | 3.5 (3.4-3.5) | 4.1 (4.0-4.2) | 3.4 (3.4-3.5) | 3.4 (3.3-3.5) | 1.01 (1.01-1.01) |
| *Clostridioides difficile/C. toxins* | 0.4 (0.4-0.4) | 0.1 (0.1-0.1) | 0.1 (0.1-0.1) | 0.1 (0.1-0.1) | 0.1 (0.1-0.2) | 0.1 (0.1-0.1) | 0.2 (0.1-0.2) | 0.1 (0.1-0.2) | 0.1 (0.1-0.1) | 0.1 (0.1-0.1) | 0.2 (0.2-0.2) | 0.2 (0.1-0.2) | 1.06 (1.05-1.07) |
| **Other tests** |  |  |  |  |  |  |  |  |  |  |  |  |  |
| Nucleic acid amplification tests | 21.8 (21.7-21.9) | 3.4 (3.3-3.5) | 3.0 (2.9-3.1) | 3.2 (3.1-3.3) | 4.6 (4.4-4.7) | 4.3 (4.2-4.4) | 5.8 (5.7-5.9) | 6.8 (6.7-6.9) | 9.5 (9.3-9.6) | 15.2 (15.0-15.3) | 10.5 (10.3-10.6) | 17.9 (17.7-18.1) | 1.21 (1.21-1.21) |

**Abbreviations: aRR-** adjusted rate ratio, **CI**- confidence interval, **EENT**- Eye, Ear, Nose, Throat

**Supplementary Table 4. Age and sex standardised number of any microbiology tests and specific tests/100 resident-years among older Australians in long-term care facilities, overall and in selected study years between 2009 and 2019 (95%CI)**

| **Microbiology tests** | 2009-2019 | 2009 | 2010 | 2011 | 2012 | 2013 | 2014 | 2015 | 2016 | 2017 | 2018 | 2019 | aRR 95%CI |
| --- | --- | --- | --- | --- | --- | --- | --- | --- | --- | --- | --- | --- | --- |
| Any microbiology tests | 196.3 (195.6-196.9) | 166.8 (166.1-167.6) | 168.3 (167.6-169.0) | 173.1 (172.4-173.8) | 183.5 (182.7-184.2) | 193.4 (192.6-194.1) | 201.5 (200.7-202.3) | 201.9 (201.2-202.7) | 206.6 (205.8-207.3) | 218.8 (218.0-219.6) | 206.8 (206.0-207.5) | 224.2 (223.4-225.0) | 1.03 (1.03-1.03) |
| **Tests grouped by body system** | | | | | | | | | | | | | |
| Urine | 133.0 (132.5-133.5) | 118.4 (117.8-119.1) | 120.7 (120.0-121.3) | 121.6 (121.0-122.2) | 126.4 (125.8-127.0) | 135.2 (134.5-135.8) | 138.9 (138.3-139.5) | 138.7 (138.1-139.3) | 139.5 (138.9-140.2) | 138.7 (138.1-139.3) | 138.6 (138.0-139.2) | 141.1 (140.5-141.7) | 1.02 (1.01-1.02) |
| Skin/superficial | 19.9 (19.7-20.0) | 18.4 (18.2-18.7) | 18.0 (17.8-18.3) | 19.3 (19.1-19.5) | 19.8 (19.6-20.1) | 20.3 (20.0-20.5) | 20.6 (20.4-20.8) | 20.7 (20.4-20.9) | 20.2 (19.9-20.4) | 20.0 (19.7-20.2) | 20.4 (20.2-20.6) | 20.6 (20.4-20.9) | 1.01 (1.01-1.01) |
| Faecal | 6.9 (6.9-7.0) | 6.2 (6.1-6.3) | 5.9 (5.7-6.0) | 6.2 (6.0-6.3) | 7.1 (7.0-7.3) | 7.1 (7.0-7.3) | 7.4 (7.2-7.5) | 6.6 (6.5-6.8) | 7.2 (7.0-7.3) | 8.2 (8.1-8.4) | 6.7 (6.6-6.8) | 7.5 (7.4-7.6) | 1.02 (1.02-1.02) |
| Respiratory | 3.6 (3.5-3.6) | 2.5 (2.4-2.6) | 2.6 (2.5-2.7) | 2.9 (2.8-3.0) | 3.4 (3.3-3.5) | 3.5 (3.4-3.6) | 3.8 (3.7-3.9) | 4.1 (4.0-4.2) | 4.2 (4.1-4.3) | 4.3 (4.1-4.4) | 3.7 (3.6-3.8) | 3.9 (3.8-4.0) | 1.04 (1.04-1.05) |
| Genital | 2.3 (2.2-2.3) | 2.0 (1.9-2.1) | 2.0 (1.9-2.1) | 2.1 (2.0-2.1) | 2.2 (2.2-2.3) | 2.3 (2.2-2.4) | 2.4 (2.3-2.4) | 2.4 (2.3-2.5) | 2.4 (2.3-2.5) | 2.3 (2.2-2.4) | 2.4 (2.3-2.4) | 2.4 (2.4-2.5) | 1.02 (1.02-1.02) |
| EENT | 2.7 (2.7-2.7) | 2.2 (2.1-2.3) | 2.2 (2.1-2.3) | 2.2 (2.1-2.3) | 2.4 (2.3-2.5) | 2.4 (2.3-2.5) | 2.8 (2.7-2.9) | 2.7 (2.6-2.8) | 2.8 (2.7-2.9) | 3.4 (3.3-3.5) | 2.8 (2.7-2.8) | 3.6 (3.5-3.7) | 1.05 (1.05-1.05) |
| Blood | 0.9 (0.9-0.9) | 0.7 (0.7-0.8) | 0.7 (0.7-0.8) | 0.8 (0.8-0.9) | 0.9 (0.8-0.9) | 0.9 (0.8-0.9) | 0.9 (0.8-0.9) | 0.9 (0.9-1.0) | 0.9 (0.8-0.9) | 1.1 (1.0-1.1) | 1.0 (1.0-1.1) | 1.1 (1.0-1.1) | 1.04 (1.03-1.04) |
| **Tests targeting specific pathogens** | | | | | | | | | | | | | |
| *Cryptosporidium* & *giardia* | 5.4 (5.3-5.4) | 4.5 (4.4-4.6) | 4.3 (4.1-4.4) | 5.0 (4.9-5.1) | 6.0 (5.9-6.1) | 5.9 (5.8-6.0) | 6.1 (5.9-6.2) | 5.4 (5.3-5.5) | 5.2 (5.1-5.4) | 6.3 (6.2-6.4) | 5.2 (5.1-5.3) | 4.9 (4.8-5.1) | 1.01 (1.01-1.01) |
| *Clostridioides difficile and C.toxin* | 0.2 (0.2-0.2) | 0.1 (0.1-0.1) | 0.1 (0.1-0.1) | 0.1 (0.1-0.1) | 0.2 (0.2-0.2) | 0.2 (0.1-0.2) | 0.2 (0.2-0.3) | 0.2 (0.2-0.2) | 0.2 (0.2-0.2) | 0.2 (0.1-0.2) | 0.2 (0.2-0.3) | 0.2 (0.2-0.2) | 1.07 (1.05-1.08) |
| **Other tests** |  |  |  |  |  |  |  |  |  |  |  |  |  |
| Nucleic acid amplification tests | 13.4 (13.3-13.4) | 5.9 (5.7-6.0) | 5.3 (5.2-5.4) | 5.6 (5.5-5.8) | 7.6 (7.4-7.8) | 7.5 (7.3-7.6) | 9.8 (9.6-10.0) | 11.2 (11.0-11.4) | 15.2 (15.0-15.4) | 25.1 (24.8-25.4) | 16.9 (16.7-17.1) | 30.4 (30.1-30.7) | 1.21 (1.21-1.21) |

**Abbreviations: aRR**: adjusted rate ratio, **CI**: confidence interval, **EENT**: Eye Ear Nose Throat

**Supplementary Table 5. Number of long-term care facilities where the proportion of residents who received the microbiology test was below, within or above the 95% confidence interval around the population mean for the funnel plots showing adjusted facility level variation in 2019**

| **Microbiology testing** | **Population mean** | **N (%) of facilities below the lower 95% CI** | **N (%) of facilities within 95% CI range** | **N (%) of facilities above the upper 95% CI** |
| --- | --- | --- | --- | --- |
| Any microbiology tests | 56.6% | 590 (21.4) | 1,614 (58.6) | 551 (20.0) |
| Urine tests | 42.3% | 639 (23.2) | 1,638 (59.5) | 478 (17.4) |
| Skin/superficial tests | 10.1% | 743 (27.0) | 1,748 (63.4) | 264 (9.6) |
| Faecal tests | 4.9% | 899 (32.6) | 1,658 (60.2) | 198 (7.2) |
| *Cryptosporidium* & *Giardia tests* | 3.2% | 1,253 (45.5) | 1,319 (47.9) | 183 (6.6) |
| Nucleic acid amplification tests | 10.4% | 996 (36.2) | 1,286 (46.7) | 473 (17.2) |

***Abbreviation****: CI* *- confidence interval*

*Overall, and top 5 most common tests were examined.*

**Supplementary Figure 1. Cohort selection**

547,067 individuals from 3,484 LTCFs

Individuals who did not identify as Aboriginal or Torres Strait Islander who accessed permanent long-term care between 1 January 2009 and 31 December 2019 (n=777,300)

**Excluded** **(n=230,233)**

- Individuals who held Department of Veterans ‘Affairs concession cards (n= 124,710)
- Individuals who entered permanent long-term care before 1^st^ January 1990 (n= 139)
- Individuals who accessed permanent long-term care for < 100-days in their first episode within the study period (n=90,179)
- Individuals aged < 65-years or > 105-years at the time they entered permanent long-term care (n=12,792)
- Individuals who received respite care for > 120 days before entering permanent long-term care (n=2,413)

**Supplementary Figure 2. Annual trends in crude proportion of microbiology testing stratified by a) sex, b) dementia, and c) diabetes, 2009-2019.**

1. **Sex**
2. **Dementia**


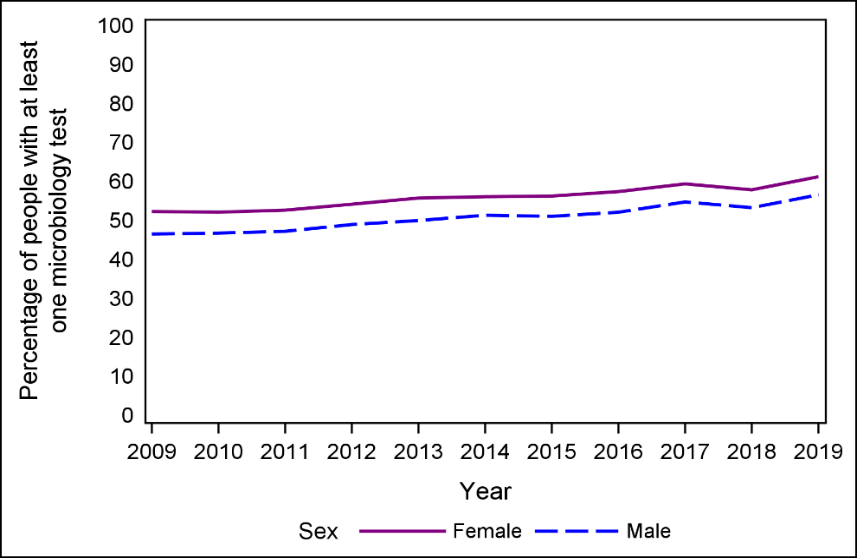

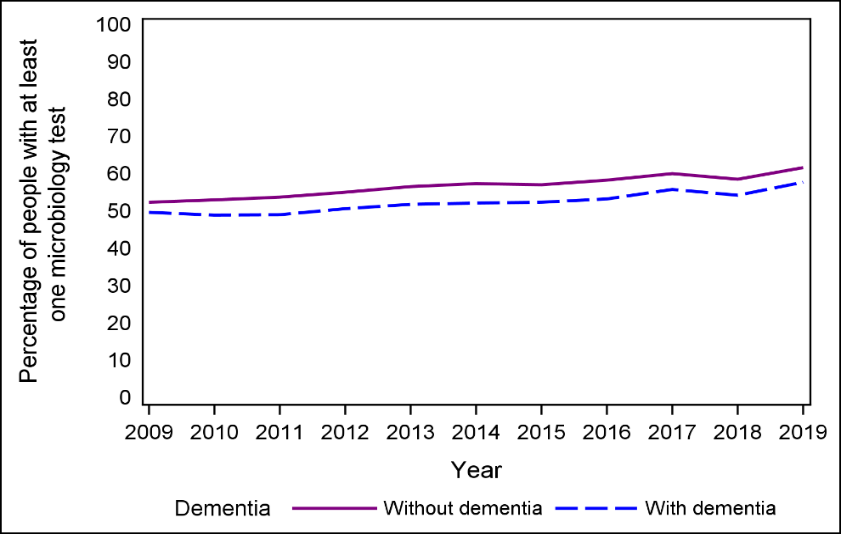


1. **Diabetes**


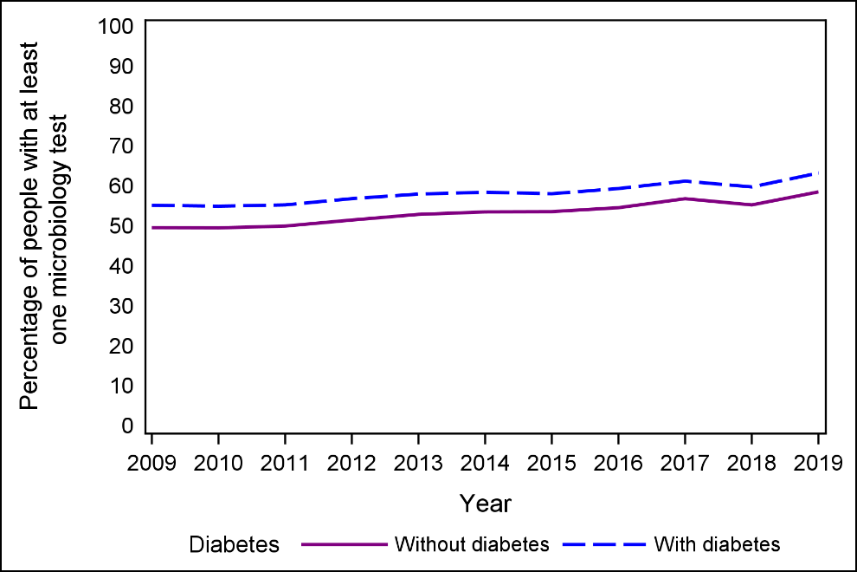


**Supplementary Figure 3. Annual trends in crude proportion of urine testing stratified by sex, 2009-2019.**


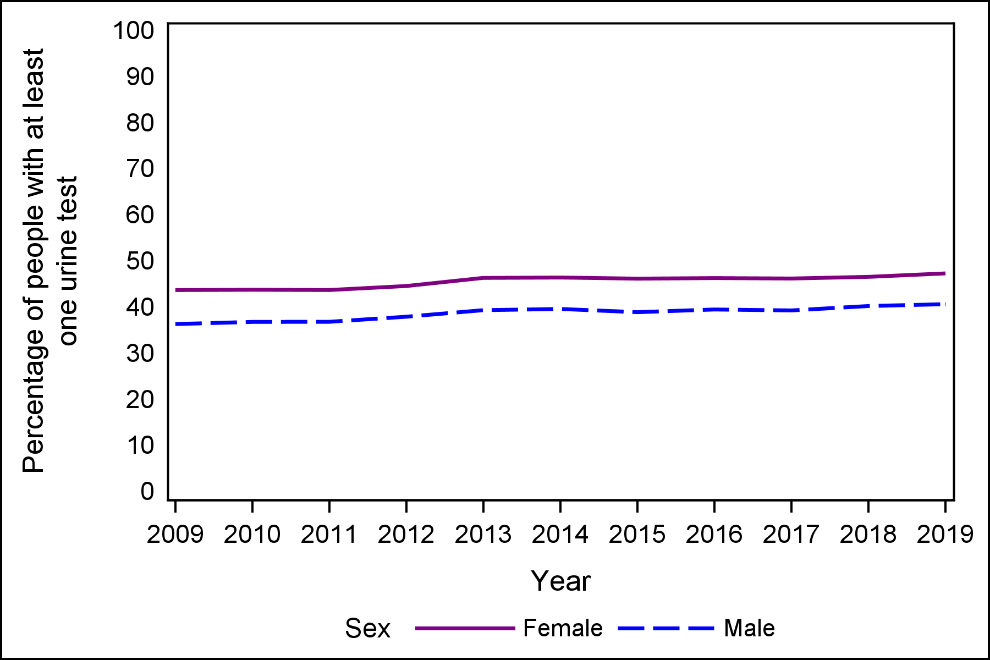


**Supplementary Figure 4**. Heat map displaying the percentage of microbiology tests performed at baseline in 2019 (shown in the rows) that were accompanied by the test shown in the column within the (a) seven next and (b) fourteen days

1. **Within seven days**

**(b)** **Within** **fourteen** **days**


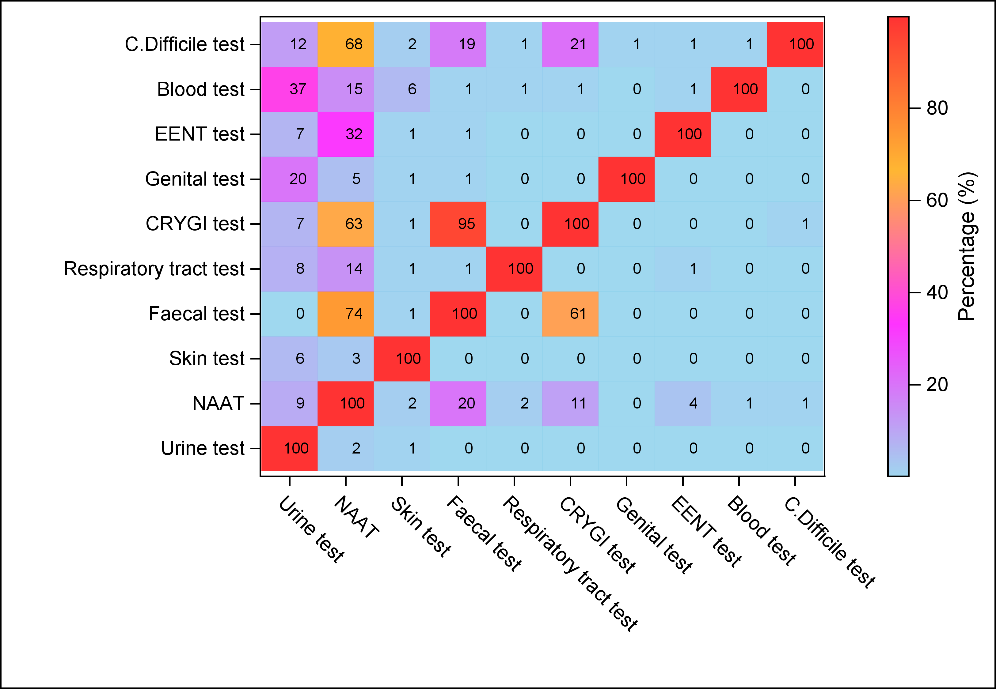

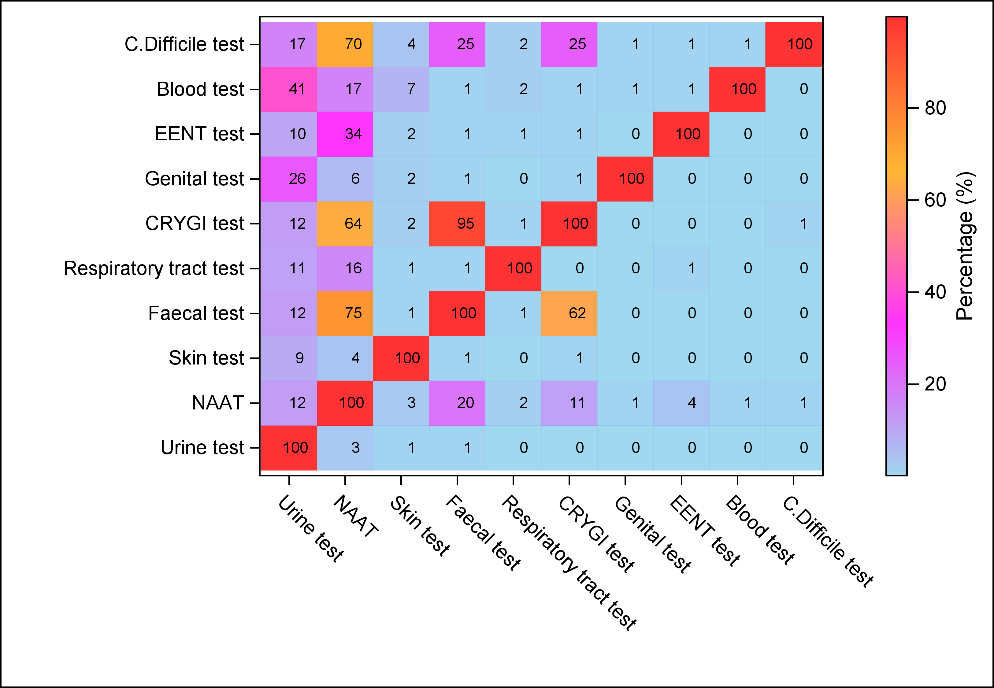


*This figure is read along the rows from left to right (e.g., 2% (3,756/211,565) of urine tests were conducted with nucleic acid amplification tests (NAAT) and 1% (1,583/211,565) are conducted with a skin/superficial test within seven days)*

***Abbreviation****:* ***CRYGI*** *- Cryptosporidium and Giardia;* ***EENT****- Eye Ear Nose Throat;* ***NAAT*** *- Nucleic acid amplification tests*

*Total number of each type of test in 2019: urine test = 211,565, NAAT = 55,630, skin/superficial tests = 31,340,*

*respiratory tests = 5,979, faecal tests = 11,268, Cryptosporidium and Giardia = 7,433, genital tests = 3,578, EENT tests = 5,434, blood tests = 1,616, Clostridioides difficile and C.toxin = 336*

**Supplementary Figure 5. Funnel plot displaying facility variation in the adjusted proportion of microbiology testing in 2019.**

**(b) Cryptosporidium/giardia tests**

**(a) Skin/superficial tests**


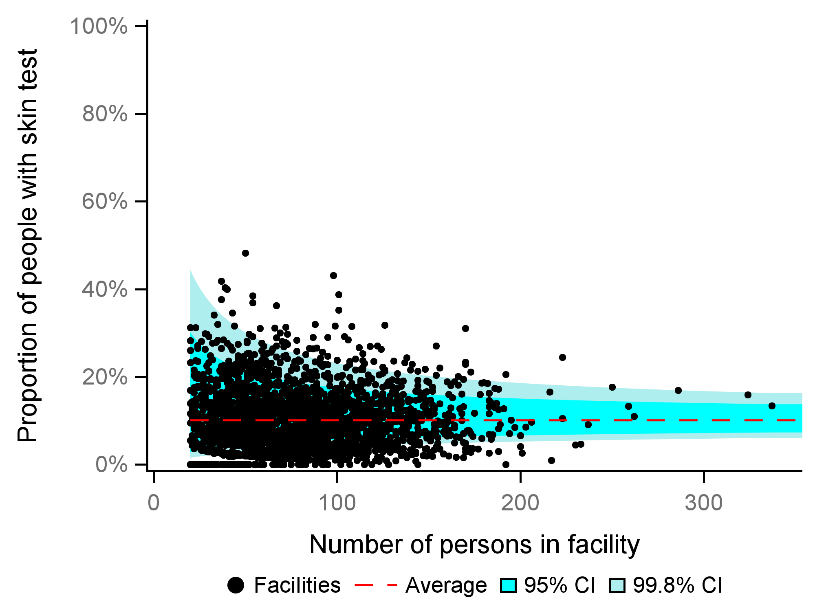

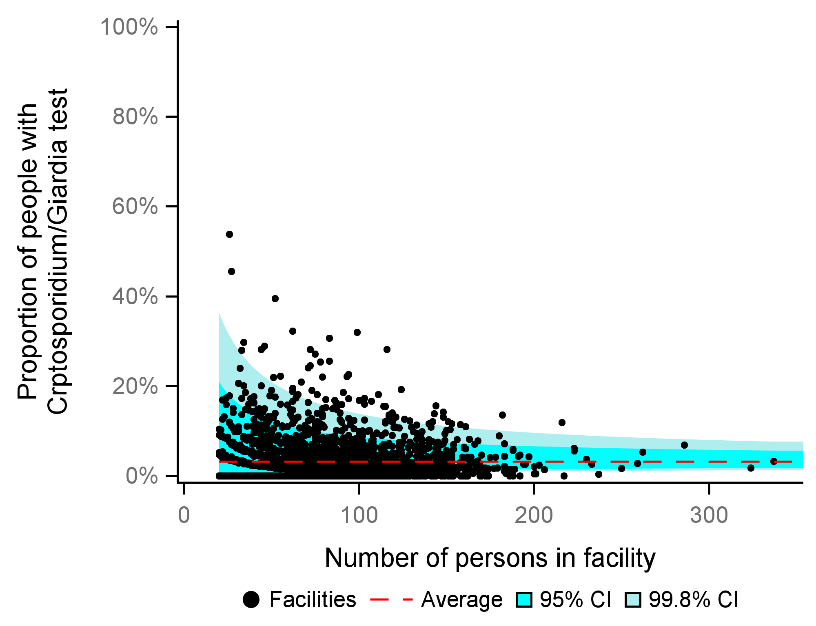

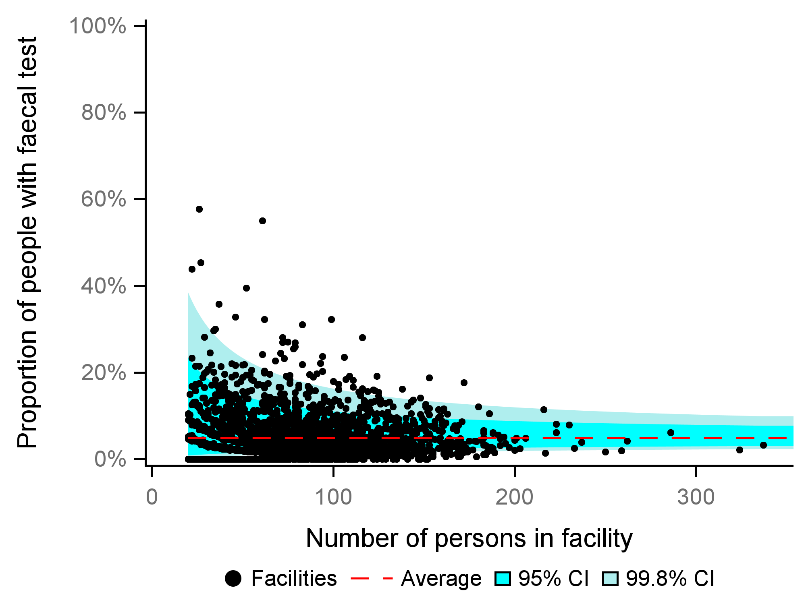

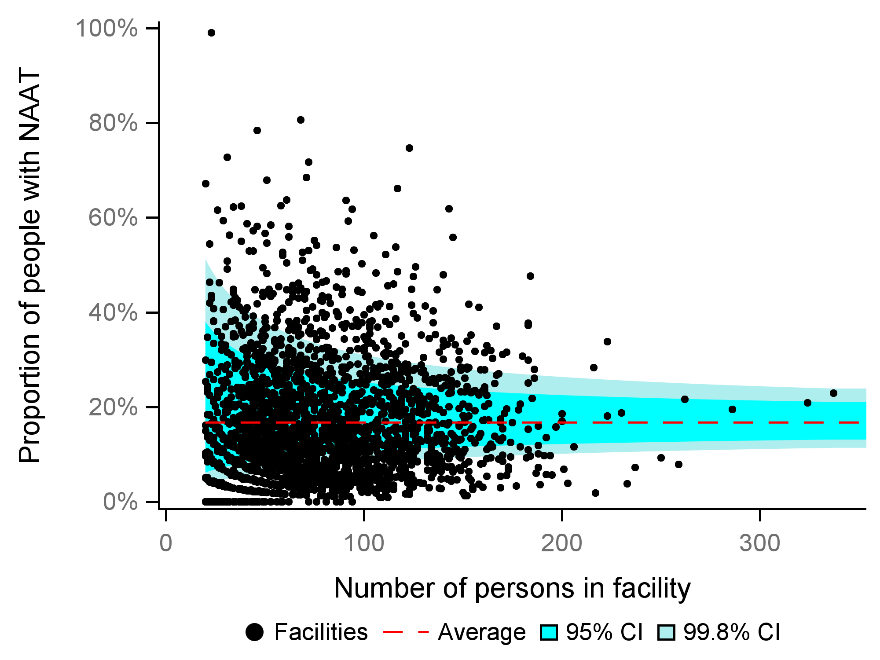


**(d) Nucleic acid amplification tests**

**(c) Faecal testing**
